# Advancements in Whole-Genome Sequencing Protocols: A Decade of In-House Operations and Quality Controls at the Tohoku Medical Megabank

**DOI:** 10.31662/jmaj.2025-0159

**Published:** 2025-10-03

**Authors:** Fumiki Katsuoka, Junko Kawashima, Shu Tadaka, Akihito Otsuki, Yasunobu Okamura, Takafumi Suzuki, Takanori Hidaka, Kazuki Kumada, Tohoku Medical Megabank Project Study Group, Fuji Nagami, Atsushi Hozawa, Shinichi Kuriyama, Nobuo Fuse, Kengo Kinoshita, Masayuki Yamamoto

**Affiliations:** 1 Department of Integrative Genomics, Tohoku Medical Megabank Organization, Tohoku University, Sendai, Japan; 2 Clinical Sequencing Group, Advanced Research Center for Innovations in Next-Generation Medicine, Tohoku University, Sendai, Japan; 3 Clinical Biobank Group, Advanced Research Center for Innovations in Next-Generation Medicine, Tohoku University, Sendai, Japan; 4 Department of Public Relations and Planning, Tohoku Medical Megabank Organization, Tohoku University, Sendai, Japan; 5 Planning and Promoting Group, Advanced Research Center for Innovations in Next-Generation Medicine, Tohoku University, Sendai, Japan; 6 Department of Preventive Medicine and Epidemiology, Tohoku Medical Megabank Organization, Tohoku University, Sendai, Japan; 7 Disaster Public Health Lab, International Research Institute of Disaster Science, Tohoku University, Sendai, Japan

**Keywords:** population genomics, genome cohort, whole-genome sequencing

## Abstract

Population-scale human whole-genome sequencing (WGS) projects are ongoing worldwide. At a time when such large-scale genome projects were uncommon, the Tohoku Medical Megabank Project initiated genome analysis of the general population in Japan, aiming to build a foundation for personalized medicine and prevention. Recently, we have completed the WGS of 100,000 participants, and research utilizing this genomic foundation is in progress. Early in the project, we realized that standard protocols were not always suitable for large-scale sequencing, necessitating the development of optimized operations and quality control methods. To accommodate various sequencing platforms and adapt protocols to the scale of analysis, we have continuously refined our methods. As multiplex sequencing analysis became standard, we aimed to ensure uniform data quantity across samples. With the advent of large-scale analyses, streamlining operations has also been a critical focus. In this paper, we share the details of our WGS operations and quality control methods developed over a decade, highlighting the unique methods and know-how we have established.

## Introduction

When the Human Genome Project (HGP) was initiated, Sanger sequencing was the primary technology employed for deoxyribonucleic acid (DNA) sequencing, and its completion required more than a decade ^(1)^. A key outcome of the HGP was the establishment of the reference genome ^(2)^. The reference genome, in conjunction with the advent of next-generation sequencing (NGS) technologies, has significantly accelerated research in human genomics. Indeed, these technological advancements have revolutionized our ability to conduct large-scale genetic studies, enhance our understanding of human genetic variation, and drive forward personalized medicine and genomic research.

The current standard NGS protocol is the short-read sequencing. This technology reads DNA sequences as short as a few hundred base pairs (bp), typically around 150 bp. Thus, it relies on the reference genome to locate reads within the genome and determine whether they match the reference entirely or exhibit variations. Due to the limited read length, this technology is not particularly adept at detecting so-called structural variations, such as insertions/deletions (indels) exceeding 50 bp. Nonetheless, for the detection of single-nucleotide polymorphisms (SNPs) and short size indels, which are the most abundant variations in the human genome, the short-read NGS has been serving as an essential foundation. Currently, one single short-read NGS machine allows us to conduct whole-genome sequencing (WGS) of up to 150 human samples simultaneously, with a turnaround time of 2 days. This enables population-scale genome sequence projects, such as the United Kingdom (UK) Biobank ^(3)^, All of Us project ^(4)^, Precision Health Research, Singapore (PRECISE) ^(5)^, and Tohoku Medical Megabank (TMM) Project in Japan.

The TMM Project is a prospective genome cohort that began in 2013, aiming to realize personalized medicine and healthcare in Japan ^(6)^. The project has recruited 157,000 participants from the general population for long-term surveys, with their invaluable cooperation ^(7)^. About 84,000 participants were recruited for a community cohort study mainly at annual health examination venues operated by local governments. Additionally, approximately 73,000 participants have joined a Birth-and-Three-Generation cohort study, where pregnant mothers initially enrolled at obstetrics clinics, followed by their newborn babies, cooperating spouses, babies’ siblings, and both the mother’s and father’s parents (grandparents from the child’s perspective). These cohort studies conduct follow-up assessments of their health status approximately every 5 years.

We initiated a WGS project alongside the start of the cohort survey in 2013. Since large-scale WGS projects were not common at that time, the process was largely exploratory. As available methods were not necessarily suitable for large-scale projects, we often needed to develop our own methods. Through such continuous improvements, we finished the WGS of 100,000 samples in 2024. With this achievement, in this paper, we share our experiences with population-scale WGS, covering aspects such as sample handling, sequencing platforms, protocols, tips, and best practices.

## Materials and Methods

### Informed consent for genomic research

All participants provided written informed consent to contribute their de-identified biospecimens and data for research purposes. Regarding genome analysis, informed consent was obtained based on the following policy: “The genome information obtained through the project may not always be sufficiently accurate or reliable to assess your health status. Immediate disclosure of such information may cause psychological stress or lead to misunderstanding for you or your family members. Accordingly, once the necessary conditions for returning individual genomic results are fulfilled, we will contact you to confirm whether you wish to receive their results”. Details of the protocol for the return of genome information were as described ^(8), (9)^.

### DNA samples

The details of sample collection and DNA extraction were previously described ^(10)^. Briefly, buffy coat samples were aliquoted by automatic liquid handling systems and stored at −80°C until use. Genomic DNA from buffy coat was extracted using an Autopure LS DNA purification system (Qiagen) or GENE PREP STAR NA-480 (Kurabo). Genomic DNA from cord blood was isolated using QIAsymphony SP (Qiagen). When blood samples were not available, DNA was extracted from saliva collected using Oragene preservative solution (DNA Genotek). In our studies, the contamination of bacterial genomes in saliva varies between 10% and 20% (data not shown). The concentration of DNA was measured using the fluorescence dye-based Quant-iT PicoGreen double-stranded DNA (dsDNA) kit (Invitrogen), adjusted to 50 ng/μL, and stored at 4°C until use.

The biobank division has been operating under the accreditation for International Organization for Standardization (ISO) 27001:2013 and ISO 9001:2015, and recently, the accreditation for Japanese Industrial Standards (JIS) Q 20387:2023 (ISO 20387:2018), an international standard that establishes a set of requirements to ensure that biobanks operate in a manner that preserves the quality and integrity of the biospecimens.

### Library preparation

Genomic DNA was diluted to 10-20 ng/μL and fragmented using a focused-ultrasonicator LE220 (Covaris) to an average target size of 550 bp and used for sequencing library preparation. Libraries for Illumina platforms were prepared with TruSeq DNA polymerase chain reaction (PCR)-free HT sample prep kit (Illumina). For NovaSeq 6000 and X Plus (Illumina), adapters with IDT for Illumina TruSeq DNA Unique Dual indexes for 96 samples (Illumina) were used. Libraries for MGI platforms were prepared with MGIEasy PCR-Free DNA Library Prep Set (MGI Tech).

### Automation

For library preparation of the TruSeq DNA PCR-free library prep kit, Agilent Bravo automated liquid handling systems with 96 channels were used with a custom-built program. For MGI platforms, the MGI SP-960 automated sample preparation system (MGI Tech) was used with its pre-built program. Biomek NXp automated liquid handling systems with 8-channel liquid handling were used for general tasks such as transferring DNA samples from sample plates to the Covaris 96 microTUBE plates (Covaris) and making a new plate by gathering samples from multiple plates.

### Library quality control

The concentration of libraries was measured with the Qubit dsDNA HS Assay Kit (Life Technologies, ThermoFisher Scientific). The size of Illumina libraries was analyzed using either a fragment analyzer (Advanced Analytical Technologies) or a TapeStation system with ribonucleic acid (RNA) ScreenTape (Agilent). The relative concentration of libraries was determined using quantitative MiSeq (qMiSeq) methods ^(11)^ or by analyzing data from multiple runs, as described below.

### Sequencing

Libraries were sequenced on each platform according to manufacturers’ protocols. For HiSeq 2500, TruSeq Rapid paired-end (PE) Cluster Kit (Illumina) and TruSeq Rapid Sequencing by Synthesis (SBS) Kit, either v1 or v2 were used. For NovaSeq 6000, the S4 Reagent Kit, either v1 or v1.5 (Illumina), was used. For NovaSeq X Plus, either the 10B or 25B Reagent Kit (Illumina) was used. For DNBSEQ-G400 (MGI Tech), DNBSEQ-G400RS High-throughput Sequencing Set (PE150) was used. For DNB-T7 (MGI Tech), DNBSEQ-T7RS High-throughput Sequencing Set v2.0 (MGI Tech) was used. We outsourced some of the samples, which were analyzed using HiSeq X (Illumina) and NovaSeq 6000.

### Data analysis

The sequencing raw data were directly transferred to a supercomputer system that was built in our institute. Details of our informatics pipelines have been reported previously ^(12)^; here, we succinctly recapitulate the core methodology. Adhering to the Genome Analysis Toolkit (GATK) Best Practices (https://www.biorxiv.org/content/10.1101/201178v3), we align FASTQ format raw data to the reference genome sequence using Burrows-Wheeler Aligner (BWA) (https://arxiv.org/abs/1303.3997) or BWA-mem2. Alignment is followed by base-quality score recalibration before performing single-nucleotide variant (SNV)/Indel calling with GATK HaplotypeCaller. Multi-sample joint calling is executed using GATK GnarlyGenotyper, and variant filtration is accomplished with the GATK VariantQualityScore Recalibration tool. Subsequent to the variant filtration process, statistical metrics such as allele frequencies are calculated to validate WGS data. The resultant allele frequency data and quality metrics, including base count and mean coverage for each WGS sample, are made publicly available via the Web database ^(13)^.

### Sequencing data quality control

For the NovaSeq series, we consistently verified whether the loading concentration of libraries was optimal by monitoring percentage occupied and pass filter (%) on the Sequence Analysis Viewer in accordance with the manufacturer’s guidelines. Based on our experience, targeting a concentration that ensures a sufficiently high %Occupied, while allowing for a slightly lower pass filter, generally yields favorable results. We also performed FastQC (https://www.bioinformatics.babraham.ac.uk/projects/fastqc/) to ensure that the duplication rate is reasonably low and that the base balance between A and T, G and C is not significantly skewed. To assess the library insert size based on mapping information, we performed CollectInsertSizeMetrics (https://broadinstitute.github.io/picard/). We also used the onboard DRAGEN integrated into the NovaSeq X, which enables FastQC and mapping immediately after base calling, to promptly evaluate the above-mentioned quality control (QC) data.

### Sample identity verification

To detect sample mix-up, we independently performed SNP array analysis for all WGS examination participants and confirmed sample integrity by assessing genotype-level concordance with WGS data. In addition, we estimated contamination using VerifyBamID ^(14)^, which detects the presence of DNA from other individuals based on allele mismatches in aligned reads. Samples with a contamination rate of ≥3% were excluded from downstream joint genotyping analyses. A similar contamination filtering approach using VerifyBamID has been adopted in other large-scale genomic studies, including gnomAD ^(15)^. Our threshold is more stringent than the 5% cutoff adopted by gnomAD. When discrepancies were observed between self-reported familial relationships and those inferred from genomic data, the corresponding samples were excluded from analyses in which such inconsistencies could have a significant impact ^(16)^.

## Results

### Importance of WGS analysis accompanied by multi-layer data

**Figure 1.**
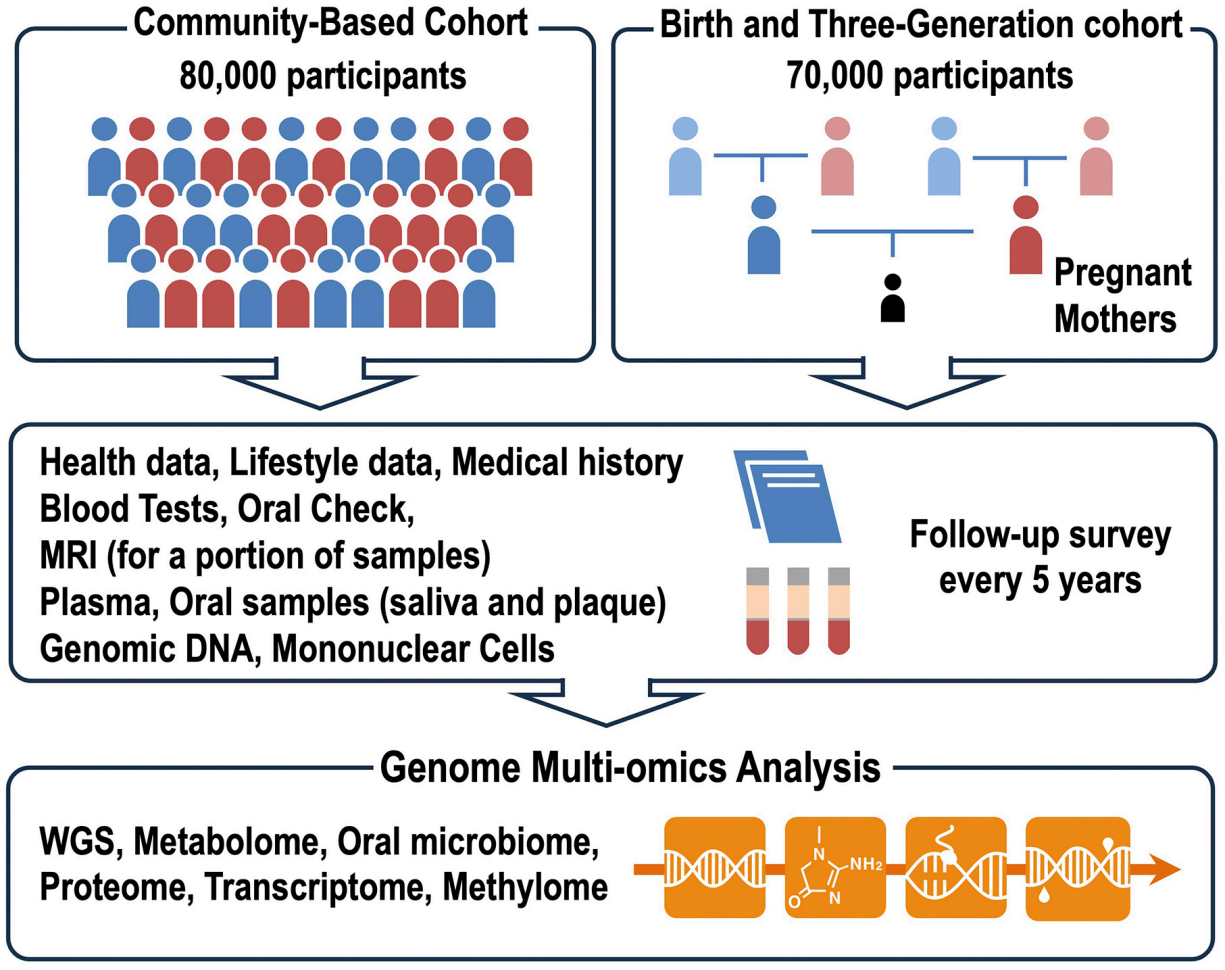
Overview of Genome and Multi-omics Analysis in the Tohoku Medical Megabank Project. The TMM Project recruited 80,000 participants for its community-based cohort and 70,000 participants for its Birth-and-Three-Generation cohorts. As illustrated, various types of samples have been collected along with data on participants’ health, lifestyle, and medical history. Follow-up surveys are conducted every 5 years. Utilizing these samples and their associated rich datasets, genome and multi-omics analyses are actively being carried out. TMM: Tohoku Medical Megabank.

The purpose of WGS analysis in cohort studies is to understand the functional contributions of genetic variations to the health status, disease onset, and disease progression. To this end, WGS data accompanied by multiple layers of real-world data and health examinations, and multi-omics data, are valuable. In the TMM Project, we strategically designed two prospective genome cohort studies, the community-based cohort ^(17)^ and the Birth-and-Three-Generation cohort ^(18)^, which include detailed baseline assessments and follow-up surveys conducted every 5 years (Figure 1). We collect a variety of health data, including lifestyle data and medical history, blood test results, oral examinations ^(19)^, and magnetic resonance imaging (MRI) data (for a subset of participants) ^(20)^. Additionally, we have stored plasma, oral samples (saliva, plaque), genomic DNA, and mononuclear cells. This design allows us to obtain WGS data accompanied by comprehensive information.

Along with WGS sequencing, we are also obtaining multi-omics data, including microarray analysis and metabolomics (Figure 1). The importance of association studies utilizing multi-omics data, along with genome variation data, can be emphasized by the integrated analysis of genome- and metabolome-derived data. These integrated analyses identified many SNVs and genomic loci affecting the metabolite levels. This analysis has been referred to as metabolome-genome wide association study ^(21)^. In our multi-omics analysis, TMM is also conducting metagenome, epigenome, and proteome data, albeit the progress is in their early stages.

### Execution of WGS analysis for participants with family relations

**Figure 2.**
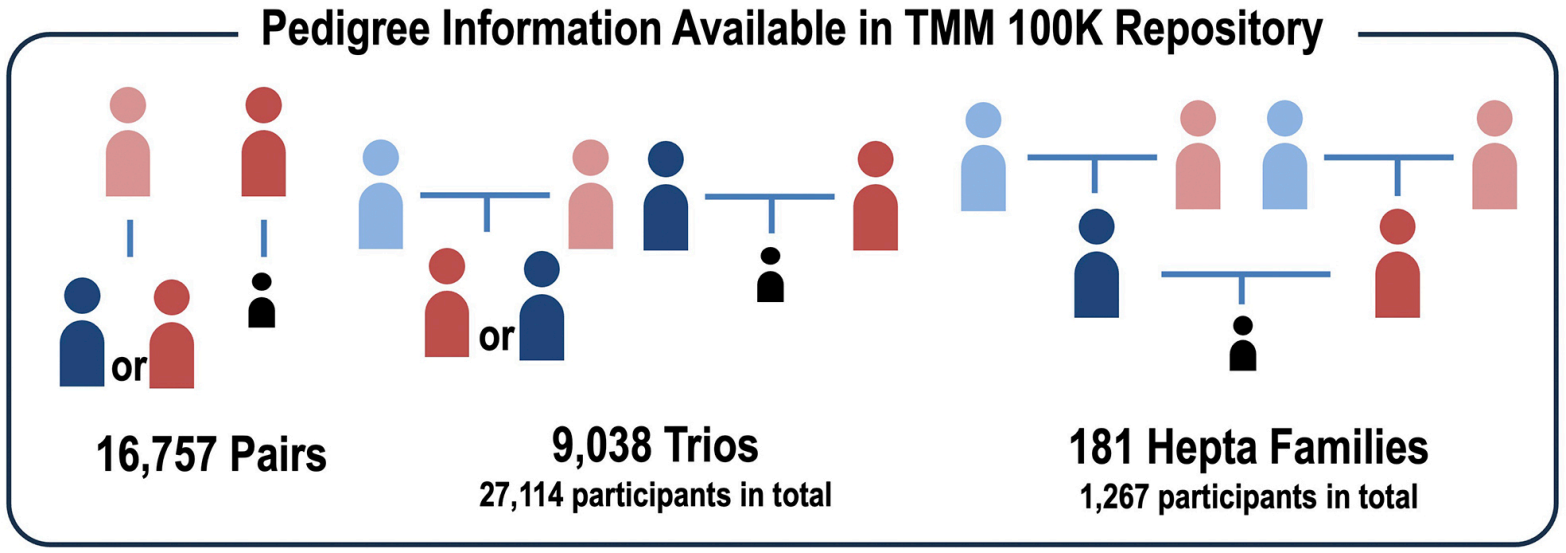
Pedigree information available in the 100K Repository. The repository contains pedigree data for 16,757 pairs and 9,038 trios, which include either parents and their child or grandparents and one parent. In addition, the repository contains 181 hepta-families, each comprising two sets of grandparents, parents, and their child. Light blue represents the grandfather, while pink represents the grandmother. Dark blue represents the father, and red indicates the mother. Black indicates the child.

At an early stage of our WGS project, we prioritized sequencing genetically independent samples, excluding close relatives. This approach aimed to efficiently catalog common haplotypes prevalent in the Japanese population and design SNP arrays optimized for the Japanese population ^(22)^. With the progression of recruitment for the Birth-and-Three-Generation cohort, we moved to the sequencing of samples derived from families. As expected, we observed a variety of family structures within the cohort. Upon completion of the 100,000-sample WGS analysis, we observed 16,757 pairs (based on mothers or grandmothers as the reference) (Figure 2). We observed 9,038 trios, including trios of grandparents and mother, grandparents and father, and parents and child. These trios include the pairs explained above. Finally, this dataset harbors 181 seven-member families, consisting of the paternal and maternal grandparents, father, mother, and child, which we refer to as hepta-families (Figure 2).

### In-house sequencing platforms: The path toward 100K WGS analysis

We began WGS in 2013 with the Illumina HiSeq 2500, which was the first NGS platform capable of performing 150 bp PE analysis on two WGS samples simultaneously. As of the time of writing this article, 150 bp PE analysis remains the de facto standard protocol for human WGS. Therefore, we have been able to implement the 150 bp or longer PE analysis in our sequencing protocol throughout the project.

**Figure 3.**
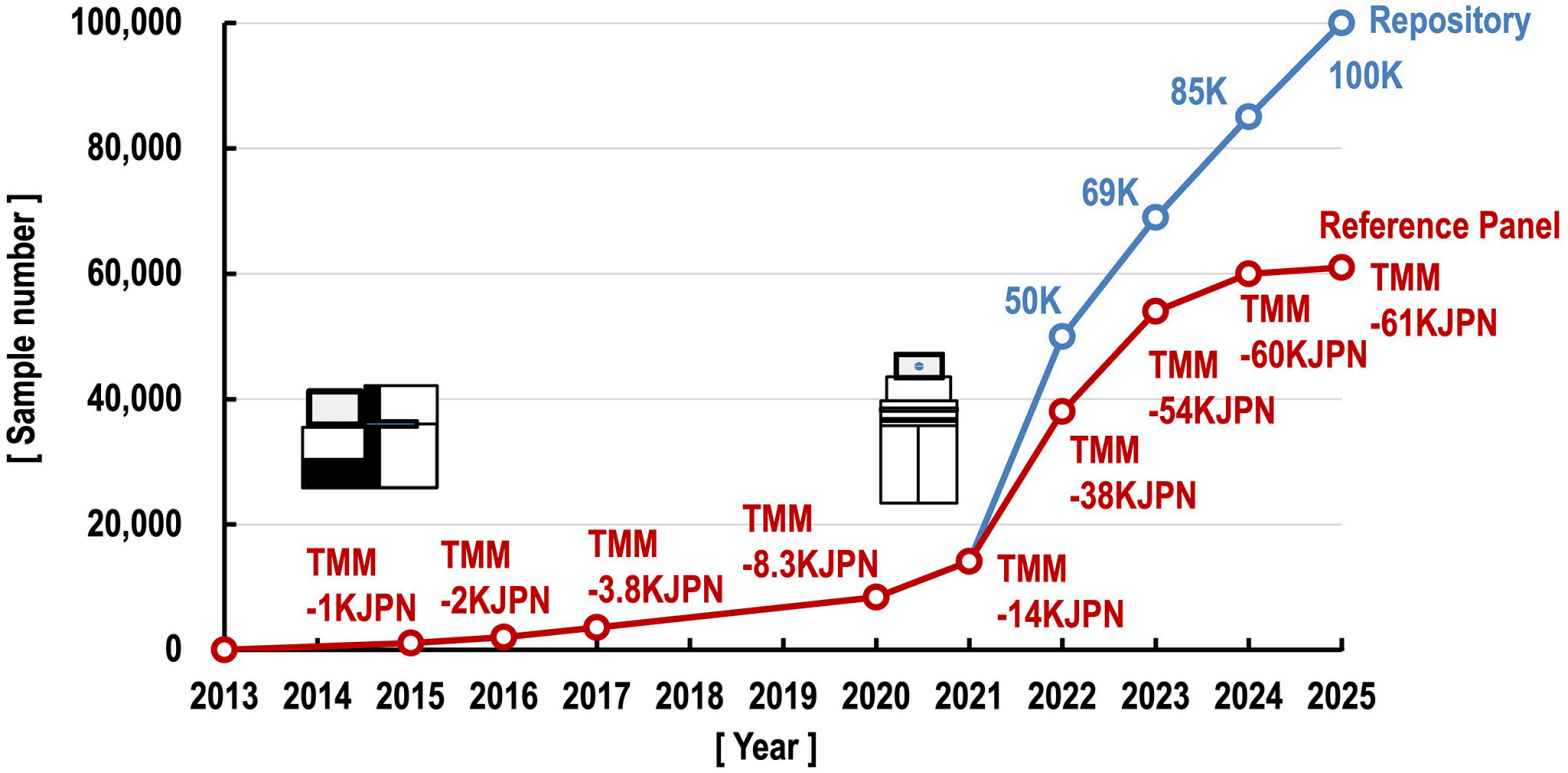
The annual progress of the TMM WGS project. The TMM genome reference panels have been continuously released since 2015 (red), and the TMM repositories, including samples related to family pedigrees, have been available since 2022 (blue). The schematic diagram on the left shows the HiSeq 2500, which was used at the project’s inception, while the diagram on the right shows the NovaSeq 6000, which accelerated the project. In March 2024, we achieved the milestone of completing the WGS of 100,000 participants. Informatics work is underway to release a new reference panel TMM-61KJPN, and a repository based on this dataset in 2025. TMM: Tohoku Medical Megabank; WGS: whole-genome sequencing.

Using the HiSeq 2500, we completed WGS of 1,070 samples and released the reference panel under the name of TMM-1KJPN, which provides a catalog of variants with allele frequencies found in the general Japanese population (Figure 3). At the time in 2015, our data set was among the largest analyzed at a high depth (Table 1). We then released the TMM-2KJPN reference panel based on 2,049 samples in 2016, the TMM-3.5KJPN reference panel on 3,554 samples in 2017, and the TMM-8.3KJPN reference panel on 8,380 samples in 2020 (Figure 3).

**Table 1.** The list of WGS Projects Conducted around 2015.

Genome projects	Sample number	Average coverage	Sample number with over 30×	Publication date
ToMMo, Japan (this project)	1,070	32.4×	1,070	August 2015^a^
deCODE, Iceland	2,636	20×	909	May 2015^b^
1000 Genome project phase 3	2,504	7.4×	453	September 2015^c^
UK10K project	3,781	7×	0	September 2015^d^
SardiNIA project, Italy	2,120	4×	0	September 2015^e^
BBMRI, The Netherlands	750	12×	0	February 2014^f^
African Genome Variation	320	4×	0	December 2014^g^

WGS: whole-genome sequencing. ^a^Nagasaki et al. ^(30)^. ^b^Gudbjartsson et al. ^(31)^. ^c^Sudmant et al. ^(32)^. ^d^UK10K Consortium et al. ^(33)^. ^e^Sidore et al. ^(34)^. ^f^Boomsma et al. ^(35)^. ^g^Gurdasani et al. ^(36)^.

TMM WGS project accelerated with the introduction of high-throughput sequencing platforms, i.e., the NovaSeq 6000, NovaSeq X Plus, DNBSEQ-G400, and DNBSEQ-T7 (Table 2). In particular, the NovaSeq 6000, NovaSeq X Plus, and DNBSEQ-T7 can generate 6 TB or more data at once, enabling us to sequence a large number of samples simultaneously. Capitalizing on this high output, we released TMM-14KJPN reference panel based on 14,129 samples in 2021, TMM-38KJPN reference panel based on 38,722 samples in 2022, TMM-54KJPN reference panel based on 54,302 samples, TMM-60KJPN reference panel based on 59,940 samples in 2024 (Figure 3).

**Table 2.** Sequencing Platforms and Protocols Used for the TMM 100,000 WGS Project.

Platforms	Mode	Protocol	Output	Approximate number
				of samples analyzed
HiSeq 2500	Rapid Run	162PE	97Gb × 2	3,400
		259PE	155Gb × 2	300
HiSeq X	N/A	150PE	900Gb × 2	<100
DNBSEQ-G400	FCL	150PE	540Gb × 2	1,400
DNBSEQ-T7	N/A	150PE	1.5Tb × 4	8,400
NovaSeq 6000	S4 flowcell	150PE	3Tb × 2	24,800
		151PE	3Tb × 2	4,900
		161PE	3.2Tb × 2	48,000
NovaSeq X Plus	10B flowcell	161PE	3Tb × 2	9,000
	25B flowcell	161PE	8Tb × 2	

FCL: Flow Cell Large; N/A: not applicable; PE: paired-end; TMM: Tohoku Medical Megabank; WGS: whole-genome sequencing.

Until the TMM-14KJPN reference panel, our released datasets were based on samples that excluded close relatives to estimate accurate allele frequencies in the population. However, we have sequenced more samples than those adopted in the panel. Starting with the TMM-38KJPN reference panel, we also released information on samples prior to excluding close relatives, as the sample repository data. We released the TMM-50K repository data alongside the TMM-38KJPN, the TMM-69K repository data along with the TMM-54KJPN, and the TMM-85K repository along with the TMM-60KJPN (Figure 3). As of the end of March 2024, we have finished WGS of more than 100,000 samples, which has been the goal of our WGS analysis. Data processing is currently underway to release both the TMM-100K repository and the TMM reference panel (tentatively named TMM-61KJPN) derived from this repository in 2025 (Figure 3).

Upon conducting the above sequencing analysis, we have been exploring optimal configurations to achieve the best results. As a result, we have conducted the WGS analyses by implementing various improvements, some of which have been published ^(11)^ and have been utilized in many laboratories (personal communication). In the following section, we will outline these efforts, including details of our WGS protocol and its QC measures.

### Selection of sequencing libraries and read length

Fortunately, when we began this project, library reagents compatible with the PCR-free method had just been released, allowing us to adopt this method from the beginning. A PCR-free library is considered the most favorable for WGS due to its lower amplification bias, which results in more uniform coverage. The Illumina TruSeq DNA PCR-Free Library Prep protocol offers two options for library insert size: 350 bp or 550 bp. Although the latter requires twice the amount of DNA compared to the former, we selected the 550 bp insert size in most cases. This decision was driven by the advantage of a longer insert size that enables the use of longer read lengths.

We have sequenced libraries with various read lengths due to technical and other considerations related to platforms and reagents (Table 2). As mentioned, a de facto standard protocol for human WGS is 150 bp PE sequencing. However, we have consistently aimed at the use of longer read lengths, when possible, as we anticipated that longer reads could reduce multi-mapping. For the HiSeq 2500, we selected 162 bp PE sequencing instead of 150 bp PE sequencing. This was due to the HiSeq 2500 SBS reagent for 300 cycles, which contained sufficient reagents to sequence Read 1 for 150 bp, Read 2 for 150 bp, and the index reads, totaling 325 cycles. By sequencing one sample at a time without index reads, we could allocate the reagents to Read 1 and Read 2. It was fortunate that the HiSeq control software was also capable of supporting a maximum read length of 162 bp. When the SBS V2 reagent for 500 cycles became available, we temporarily adopted 259 bp PE sequencing.

For the NovaSeq 6000, we initially employed 150 bp PE sequencing through outsourcing, following the manufacturer’s recommendation. After installing our own NovaSeq 6000 platforms, we opted for 161 bp PE sequencing. At the time of writing, the NovaSeq 6000 SBS reagent for 300 cycles includes 338 cycles, allowing us to perform 161 bp PE sequencing along with 8 bp x 2 index reads. The 161 bp PE protocol is also applicable to the NovaSeq X Plus platforms. For the DNBSEQ series, we followed the manufacturer’s recommendation, which limited us to 150 bp PE protocols for all samples.

### Determination of sequencing coverages

High-depth WGS is now standard, but at the time when this project began, it was not as popular as it is now (Table 1). Our initial sequencing platform, the HiSeq 2500, provided a rapid run mode enabling 150 bp PE sequencing with 300 million reads per flow cell. Analyzing one sample per flow cell yields ~90 Gb of data, which calculates to approximately 30× coverage for a human genome. To determine genotype without imputation, we chose the one sample per flow cell method, aiming for 30× coverage. Since we utilized 162 bp PE sequencing, we theoretically achieved coverage of 30× or higher under optimal conditions. This is one of the advantages of selecting a longer read length.

We had been targeting a mean coverage of 30×, but approximately 30,000 samples were adjusted to 20× coverage. According to a previous study examining the effect of sequence coverage on variant detection accuracy, 20× coverage maintains good accuracy compared to 30× or more ^(23)^. Therefore, considering the balance between cost and accuracy, and the need to accelerate the project, we have chosen 20× coverage for part of our samples.

### On-premises TMM supercomputer system for large-scale data analysis

The informatics part is facilitated by the on-premises TMM supercomputer system, which is specifically optimized for human genome analysis. As genome analysis demands have grown, we have updated the system three times, with the current system representing the 3rd generation (phase III system). Notably, the phase III system features a significantly high-speed storage capacity, essential for storing WGS data from a large number of samples. It equips approximately 50PB of storage space, crucial for accommodating the vast volume of data generated from our sequencing operations. To ensure a fluid and secure data handling process from the sequencing platforms to the computational analysis, all sequencing platforms are seamlessly integrated with the TMM supercomputer system via a dedicated private network. This design provides robust isolation from the public network, offering the highest level of data protection and security. Details of our informatics pipelines are provided in the methods section.

### Library QC and data balancing for a large-scale analysis with HiSeq 2500

Library QC is critical for ensuring high-quality sequencing data. Although manufacturers provide QC methods, these often lack comprehensive details, requiring users to exercise discretion. Moreover, these methods are not always suited for large-scale analyses. Consequently, we have developed QC methods tailored to each sequencing platform and protocol used in our workflows.

The HiSeq 2500 does not utilize patterned flow cells, resulting in a very narrow range of optimal library concentrations. Excessive library concentration can lead to over-clustering on the flow cells, which reduces the number of pass filter reads and degrades data quality. Conversely, low library concentration causes under-clustering, leading to reduced data yield. Therefore, precise quantification of library concentration is crucial for achieving optimal amounts of data with high quality.

**Figure 4.**
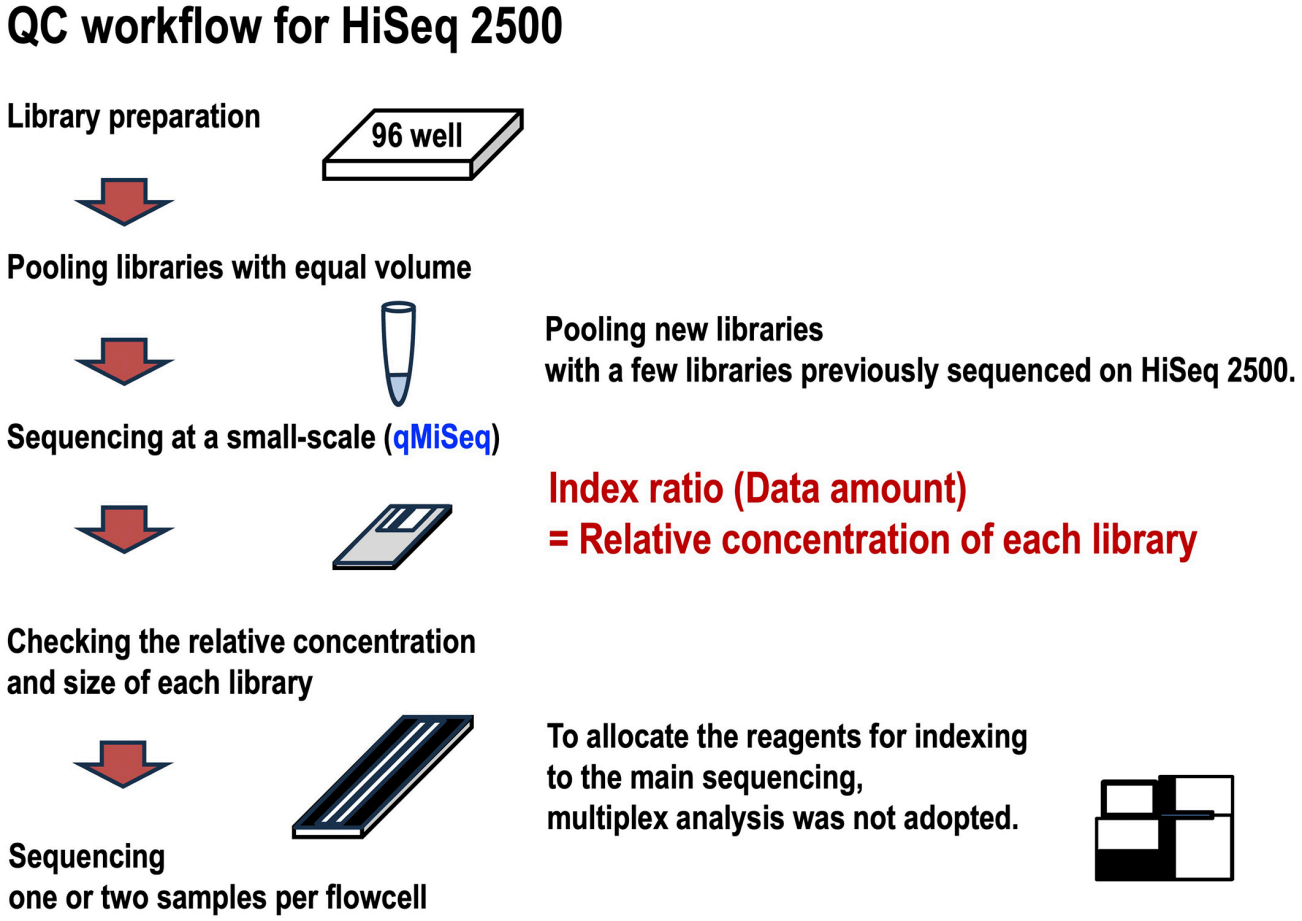
QC workflow for HiSeq 2500. Pooled new libraries, along with a few libraries previously sequenced on the HiSeq 2500, are processed through small-scale sequencing on the MiSeq to obtain the index ratio. When the new libraries are pooled in equal volumes, the index ratio reflects the relative concentration of each library. Based on this relative concentration, the run conditions for the HiSeq 2500 are determined. QC: quality control.

The manufacturer’s recommended methods for PCR-free libraries were quantitative PCR and electrophoresis-based analyses, which were unsuitable for large-scale genome analyses. Consequently, we developed our own QC method, termed qMiSeq, the details of which have been previously reported ^(11)^. This method involves mixing newly prepared libraries in equal volumes and sequencing them on a small scale using MiSeq (or any other low-throughput sequencer) and allows us to obtain relative quantification of each sample (Figure 4). By including libraries that have been previously analyzed on the HiSeq, we can finely adjust the run conditions based on their relative concentrations compared to previous samples. Additionally, the small-scale MiSeq data enables us to verify the library insert size by mapping reads to the reference genome, a task that can be performed on MiSeq using the preinstalled software.

### Library QC and data balancing for a further large-scale analysis with the NovaSeq series

The qMiSeq-based fine adjustment of library concentration was crucial for the HiSeq platforms, which offer a very narrow range of optimal concentrations. In contrast, such fine adjustment is less crucial for the NovaSeq series sequencing platforms, which allow a wide range of optimal library concentrations. Furthermore, to meet the high-throughput capacity of the NovaSeq series, simplified QC procedures are preferable.

**Figure 5.**
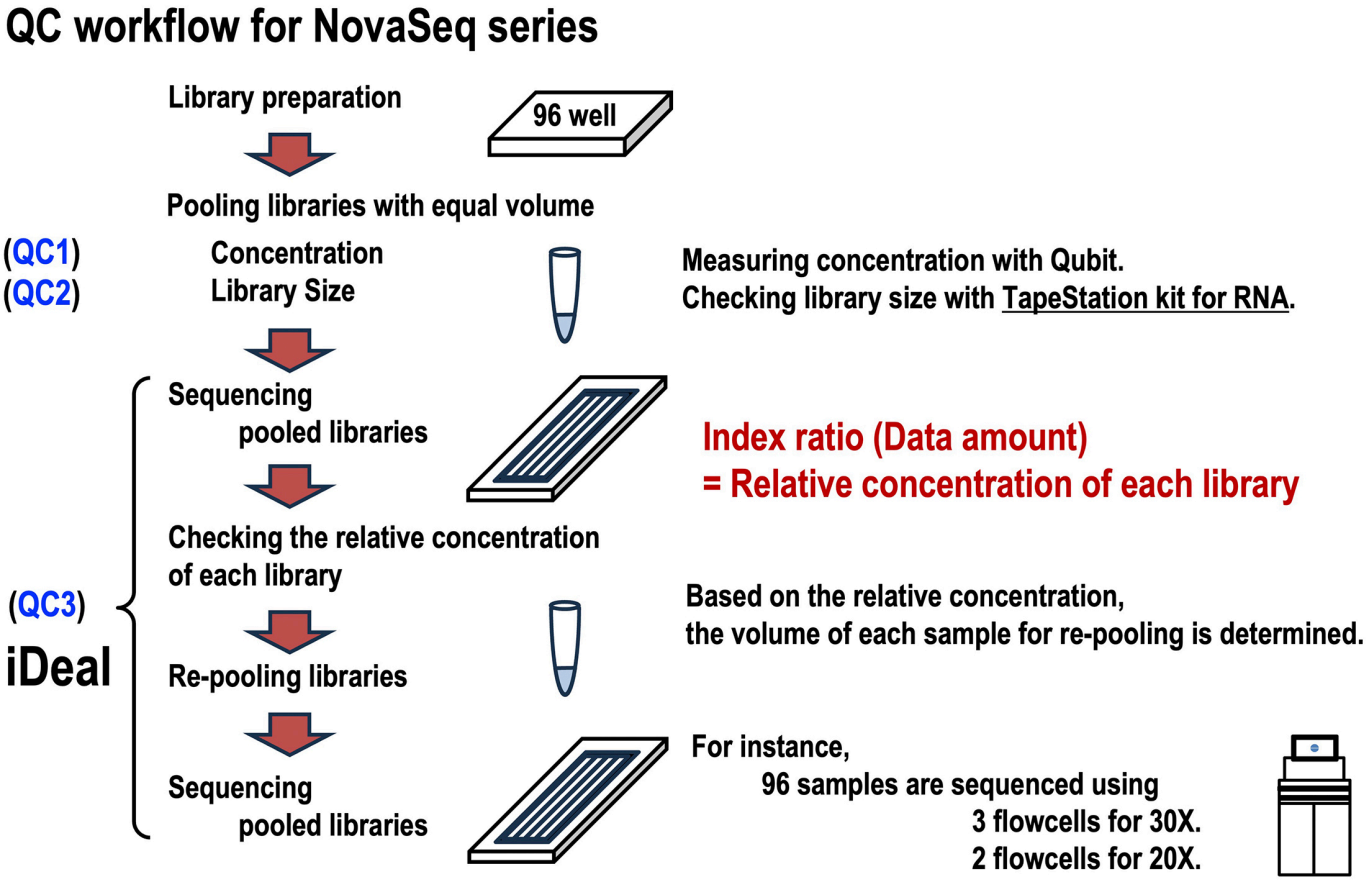
The QC workflow for the NovaSeq series. QC1 involves the measurement of the concentration of the pooled new libraries using the Qubit. QC2 is the assessment of library size using the TapeStation RNA Kit. QC3 is iDeal (initial run-based data equalization), which involves performing multiplex sequencing runs multiple times. Based on the relative concentration, the volume of each sample for re-pooling is determined. QC: quality control.

In addition, we found that the qMiSeq-based single-sample QC is not essential for multiplex-based large-scale analyses with NovaSeq series, as single-sample QC was intended to identify failures in library preparation that result occasionally from malfunctions in automated systems or from intrinsically low-quality input samples. When the failure rate is low (usually <0.1% in our facility), it is not particularly cost-effective to discard a failed sample in 96-well plate-based library preparation. Given these circumstances, we discontinued the use of qMiSeq, but adopted the following set of QC protocols, consisting of QC1, QC2, and QC3 (Figure 5).

QC1 involves the dye-based quantification of pooled libraries (Figure 5). Instead of quantifying each library sample, we measured the concentration of pooled libraries, which were prepared by mixing 96 libraries with different indexes in equal volumes. Simultaneously, we quantified the concentration of previously sequenced pooled samples. Using the relative ratios to these previously sequenced pooled samples, we calculated the total amount of pooled library DNA required for the sequencing reaction. The concentration of libraries should be considered in terms of molar concentration, which varies depending on the length of the library. Since the QC1 method assumes no significant difference in library size, we verified the library size using the QC2 method described below.

**Figure 6.**
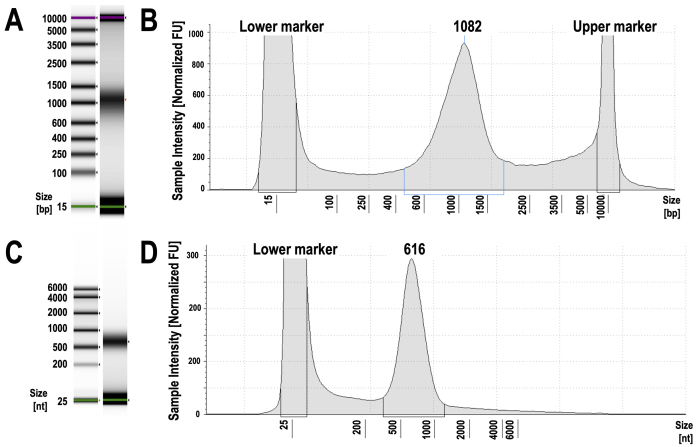
Representative data of the library size assessment using TapeStation D1000 and TapeStation RNA. With TapeStation D1000, the Illumina PCR-free library of ~600 bp migrates as a larger fragment, typically around 1000 bp or more, due to its Y-shape adapters (A and B). In contrast, with TapeStation RNA, the Illumina PCR-free library of ~600 bp migrates as expected, as the library is denatured and migrates as a single strand (C and D). PCR: polymerase chain reaction; RNA: ribonucleic acid.

QC2 refers to the size measurement of pooled libraries (Figure 5). We measured the library sizes using TapeStation reagents for RNA and confirmed that they were approximately 550 bp, as intended, with no significant differences compared to previous samples. The use of TapeStation reagents for RNA represents a key improvement for QC2. It has been observed that the Y-shaped adapter DNA used in the Illumina PCR-free library preparation kit causes libraries to migrate differently than expected. Specifically, when we examined the library size following the manufacturer’s recommendation ―i.e., using the TapeStation reagent for DNA―the result showed migration patterns exceeding 1000 bp, far longer than the expected pattern. This makes it difficult to detect size differences (Figure 6A and B). In contrast, the use of TapeStation reagent for RNA enables electrophoresis of libraries as single-stranded DNA under denaturing conditions, allowing us to measure library sizes more accurately than with the TapeStation reagent for DNA (Figure 6C and D).

It should be noted that TapeStation reagents for RNA use an RNA ladder, and the migration patterns of single-stranded DNA and single-stranded RNA differ slightly. According to a previous study, RNA ladders migrate approximately 30 nucleotides slower than DNA ladders under denaturing conditions ^(24)^. For example, the 616 bp library shown in Figure 6D corresponds to a 646 bp library, yielding an approximate insert size of 500 bp after subtracting the ~140 bp adapter sequences.

QC3 is a data volume equalization step (Figure 5). In multiplex-based high-throughput sequencing, it is critical to equalize the amount of data or sequencing depth across samples. When sequencing depth is not uniform, some samples may have low-depth data, potentially resulting in low-quality genotyping. In such cases, additional analyses will be necessary to increase the total read depth, thereby increasing overall costs. To achieve uniformity in sequencing data yield, we developed a method named “initial run-based Data equalization” (iDeal), which involves performing sequencing across multiple runs. Even when sufficient data can be obtained from a single sequencing run, we routinely conduct multiple sequencing runs using multiple flow cells.

**Figure 7.**
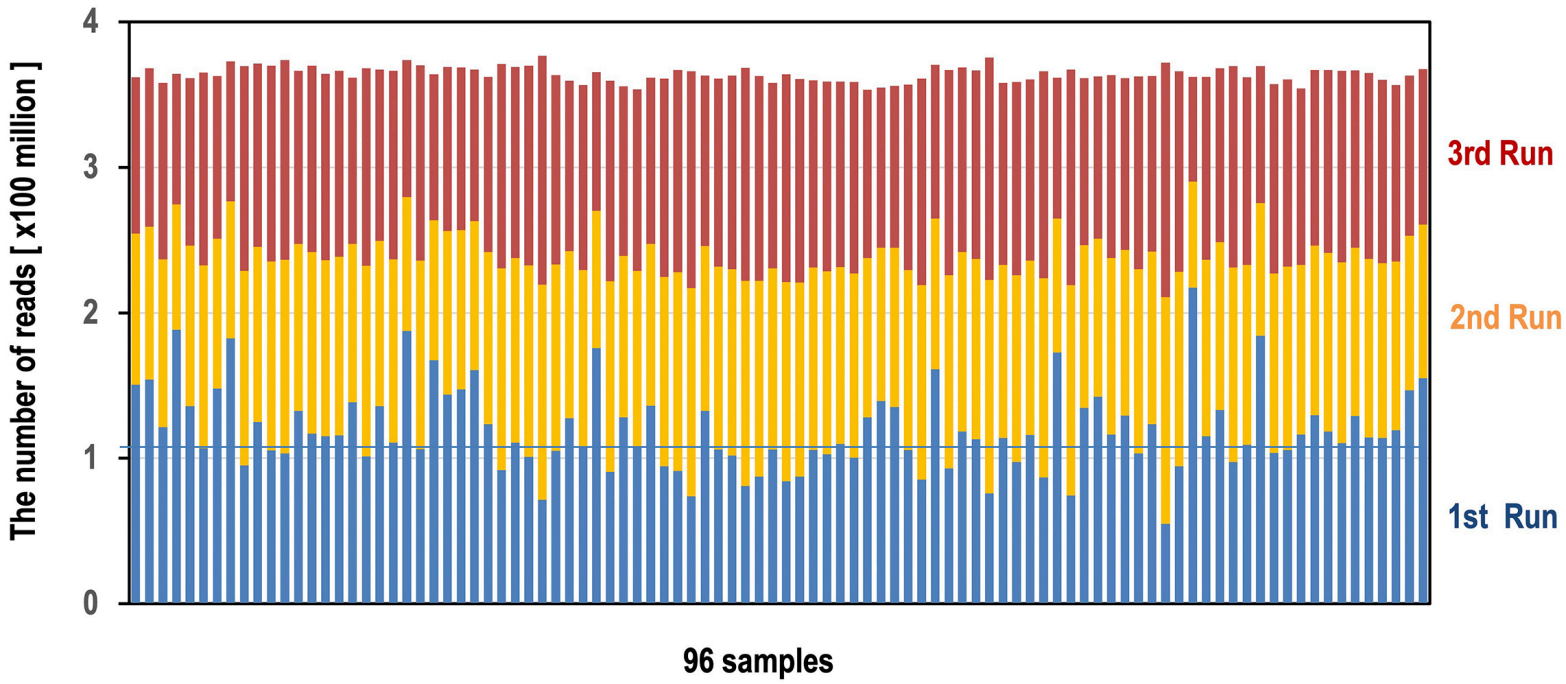
Representative data of iDeal-based sequencing. A total of 96 pooled libraries were sequenced on the NovaSeq 6000 using three S4 flow cells. In the first run, equally pooled libraries were sequenced to obtain relative concentration data for each library based on its index ratio. Subsequently, using the obtained relative concentration data, the 96 libraries were re-pooled with adjusted volumes to ensure consistent final data by sequencing with the remaining two flow cells. The number of reads from each library in the first run is shown in blue, while those from the second and third runs are shown in orange and red, respectively.

The advantage of the iDeal approach is that, based on the relative data volume of each sample obtained from the first run, we can adjust the mixing volumes of libraries in subsequent runs to achieve as even a read distribution as possible. To easily estimate relative concentrations, we perform the first sequencing with a 96-pooled library, where each sample is mixed in equal volumes, similar to the process used for qMiSeq. In the initial run, the data quantity variations between samples can exceed twofold (Figure 7, blue bar). However, by leveraging the iDeal method, we achieve well-adjusted WGS outcomes with less than a 10% variation at the end (Figure 7). If there are low-quality samples in the first run of iDeal, we exclude them and perform a second sequencing run.

### Library QC and data balancing for the DNBSEQ series

The QC process for the DNBSEQ series differs fundamentally from that of Illumina platforms ^(25)^. This difference arises from the use of cyclized libraries in the DNBSEQ series, which serve as templates for the amplification of DNA Nanoballs (DNBs) that are attached to the flow cell. Therefore, it is no longer feasible to check the library size, so we performed only dye-based quantification of the pooled libraries and the pooled DNB mixture.

We utilized the iDeal method for DNBSEQ platforms in the same way as for NovaSeq platforms. For instance, 96-sample pooled libraries were sequenced on the DNBSEQ-G400 using 16 flow cells (PE150-FCL) or sequenced on the DNBSEQ-T7 using 6 flow cells (PE150). In the case of the DNBSEQ-T7, we sequence pooled libraries using 3 flow cells for the initial round of data volume equalization. Based on the relative concentration obtained from the initial round, we sequenced the adjusted pooled libraries using 3 flow cells.

### Quality assessment with aggregated data

One of the trends revealed by the in-depth assessments of the large-set sequencing dataset is the sequence coverage profile. In the protocol targeting 30× coverage, ideally, sequenced reads would accumulate to achieve an average of 30× coverage across the genome. However, since sequenced reads are obtained randomly, the coverage from individual datasets is not uniformly 30×, making it challenging to discern the specific trends for each condition. Therefore, based on data from 1,000 samples, we calculated the mean coverage in each region and identified genomic regions that are accessible or inaccessible with each sequencer and protocol.

**Figure 8.**
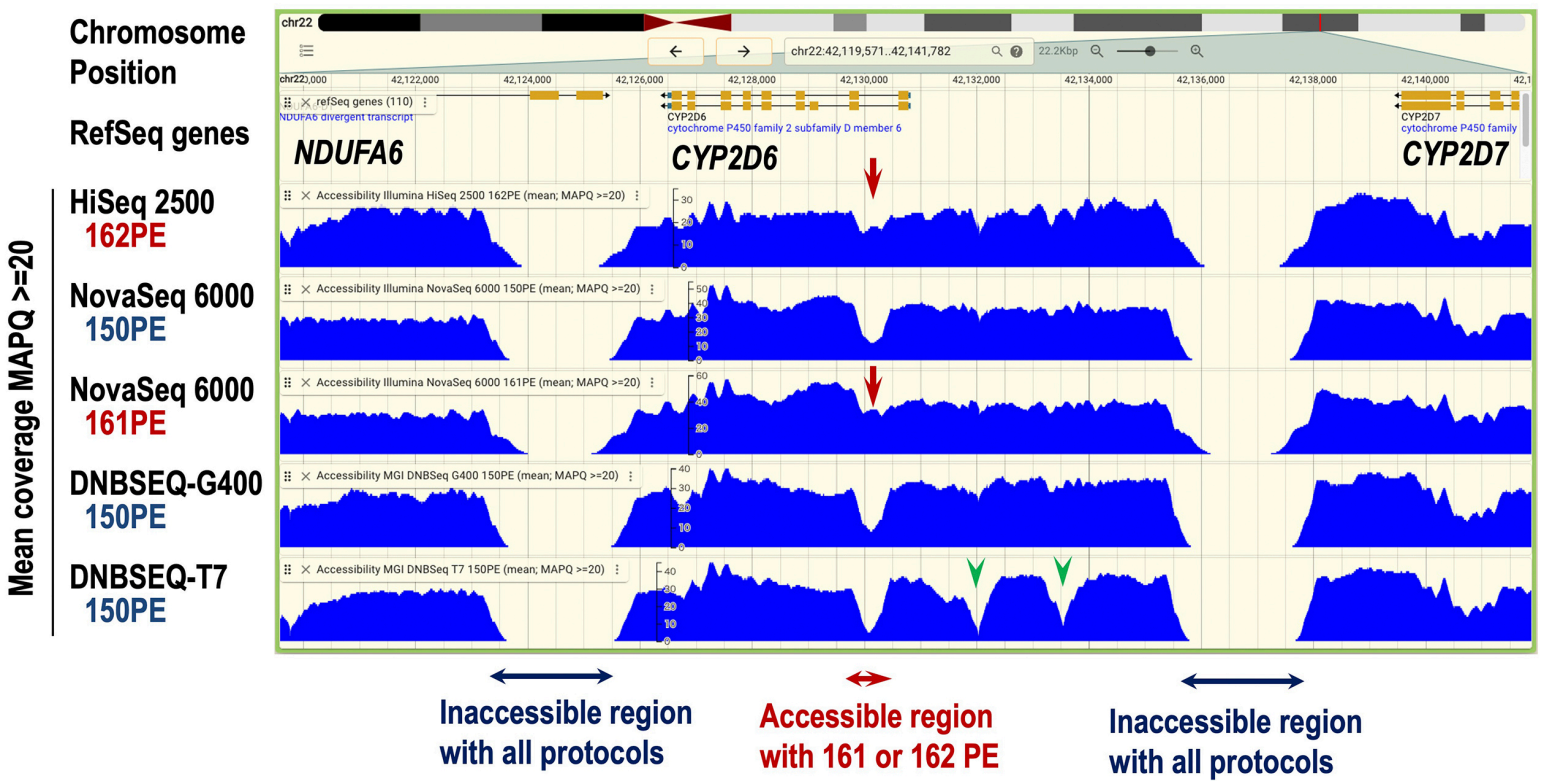
Snapshot of mean coverage data at the *CYP2D6* gene locus. The data, sourced from the jBrowser embedded in jMorp, is displayed. The chromosome position and RefSeq genes at the locus are shown above. The mean coverage, with a map quality score (MAPQ) ≥20, is calculated from the WGS data of 1000 samples sequenced on each platform and protocol shown on the left. Inaccessible regions for all protocols are indicated by blue arrows, while an accessible region with the 161 or 162 bp PE protocol is marked with red arrows. Regions where DNBSEQ-T7 shows low mean coverage are indicated by green arrowheads. PE: paired-end.

An example of the coverage data profile for the *CYP2D6* gene region is shown in Figure 8, in which certain regions remain inaccessible to all sequencers and protocols. This may be due to the insufficient read length to uniquely map the appropriate position. Importantly, however, we noticed that one of such regions is accessible by the 161 bp and 162 bp read protocols that we have incorporated, but not by the 150 bp read protocol (Figure 8, arrows). These results clearly demonstrate that the additional 11-12 bp in read length increases mapping ability. We also noticed that there are regions that are inaccessible only to DNBSEQ-T7 (Figure 8, arrowheads). Since DNBSEQ-T7 data is obtained by a PCR-free method, there may be some bias in the process due to DNB formation. This trend was not obvious with the DNB-G400 (Figure 8). The mean coverage data from the TMM WGS analyses are publicly available on the portal site, jMorp (Japanese Multi-Omics Reference Panel) ^(13)^.

## Discussion

We have provided two distinct QC protocols based on the scale of the analysis and the throughput of the equipment. In the initial phase of the TMM Project, NGS machines had much lower throughput than the current ones. To fully optimize their limited performance, we developed a method based on single-sample QC, optimized for small-scale analysis. As technology advanced, our WGS project transitioned to high-throughput machines, with most of our efforts subsequently focused on developing bulk-based protocols suitable for large-scale analysis. These efforts have enabled us to improve throughput and reduce costs while maintaining high-quality results. We believe that our experiences are adaptable to a wide range of project sizes and research objectives.

We assume that DNA sample quality can vary between projects and significantly impacts both the level of care required for library QC and the overall sequencing output. Specifically, consistent DNA sample quality, such as minimal variation in DNA concentration and the absence of DNA degradation, must be ensured. Fortunately, we were allowed to use high-quality DNA extracted by our biobank teams, which employ well-managed DNA extraction processes ^(10)^. This has contributed to simplifying library QC by enabling bulk processing and ensuring the smooth operation of large-scale analysis.

While short-read sequencing has been the global standard for more than a decade, this technology, even with current machines, faces limitations in detecting SNVs in highly repetitive regions and structural variations. These limitations are due to the read lengths, which have seen limited improvement over time. Indeed, our WGS mean coverage data clearly demonstrate that certain genomic regions remain inaccessible using current short-read platforms. To address this challenge, albeit partially, we adopted longer read lengths, such as 161 or 162 bp instead of the standard 150 bp, which improved the analysis shown in Figure 8. This adjustment, while not a complete solution, represents a step forward in overcoming the inherent limitations of short-read sequencing and can be easily applied to other projects.

Another approach we have taken to improving the accuracy of short-read sequencing is optimizing the reference genome sequence, specifically tailored to the Japanese population, as the international reference genome sequence does not represent East Asian populations. To address this, we have constructed the Japanese reference genome JG1 using genomic DNA from three volunteers and long-read sequencing platforms ^(26)^. Employing JG1 for short-read analysis in the Japanese population reduces mapping artifacts around structural variants compared to the international reference genomes GRCh38 ^(26)^. Furthermore, approximately 2% of the reads per individual that were unmapped to GRCh38 could successfully be remapped to JG1 with high mapping quality (≥20). These results indicate that JG1 recovers ethnic-specific sequences absent from GRCh38, thereby increasing both the mapping rate and the coverage of the analyzable regions. We have continued our efforts to develop newer versions of the Japanese reference genome sequences with fewer gaps. These efforts have resulted in the release of updated versions, JG2 and JG3, the latter achieving near telomere-to-telomere (T2T) quality, both of which are also made publicly available through jMorp. Detailed descriptions will be provided in forthcoming publications.

As a more direct approach to improving the quality of genome analysis, many sequencing projects have started incorporating long-read sequencing technology, which has enabled more accurate and comprehensive analysis of complex genomic regions compared to current short-read approaches. In particular, large-scale long-read sequencing projects, complementing pre-existing short-read projects, have already been initiated in several countries ^(27), (28)^, reflecting growing interest in the potential benefits of this technology. Using long-read sequencing technology, we have also sequenced 333 samples from 111 trios in our Birth-and-Three-Generation cohort, which has allowed us to create a reference panel for structural variant frequencies, verified by Mendelian inheritance error analysis ^(29)^. While this panel has proven to be a valuable resource, we acknowledge that its current size is insufficient to fully capture the spectrum of rare structural variants. We are actively working to expand the scale of this project.

One of the strengths of the TMM Project lies in its extensive baseline health examinations and long-term follow-up of participants, including MRI measurement and dental check-ups for volunteer participants ^(6)^. The TMM Project has also been conducting in-depth omics analyses, such as metabolome, transcriptome, epigenome, and metagenome analyses. These examinations enable the association studies between genome-omics data and longitudinal health records. Another characteristic of the TMM Project is that the majority of participants are of Japanese ancestry. This feature contrasts with ethnically diverse cohorts, such as the UK Biobank, All of Us, and PRECISE. We recognize the importance of establishing collaboration and data sharing with other international projects.

We acknowledge that a significant challenge ahead is the implementation of genome-based medicine into actual clinical practice. In line with this goal, we have also initiated the return of genomic results to participants, marking a step toward the practical application of personalized healthcare ^(8), (9)^. One of our limitations lies in the acquisition of precise clinical data when our participants develop diseases. To overcome this issue, the TMM biobank is conducting data acquisition from collaborating hospitals through various approaches.

### Conclusion

We provided a detailed protocol employed in the TMM 100,000 WGS analysis, including the technological and procedural improvements made over time. As a biobank established by a university, TMM has made efforts to internalize genome-omics analyses. This approach allows us to maintain control over the quality and accuracy of our data, ensuring that the results meet our research objectives. We hope that our experience can offer valuable insights for laboratories involved in WGS.

## Article Information

### Acknowledgement

First of all, we thank all the volunteers who participated in this study. We also thank all faculty and technical staff members of Tohoku Medical Megabank Organization for their assistance. We thank Prof. Yutaka Suzuki at Tokyo University, who kindly shared his knowledge and valuable experience with us. We also thank the Iwate Medical University, Iwate Tohoku Medical Megabank Organization, for their collaboration. The full list of members is available at: https://www.megabank.tohoku.ac.jp/english/a240901 for ToMMo and http://iwate-megabank.org/en/ about/departments for IMM.

### Author Contributions

This study was designed and conducted by Fumiki Katsuoka, Junko Kawashima, Shu Tadaka, Akihito Otsuki, Yasunobu Okamura, Takafumi Suzuki, Takanori Hidaka, Kazuki Kumada, Fuji Nagami, Atsushi Hozawa, Shinichi Kuriyama, Nobuo Fuse, Kengo Kinoshita, and Masayuki Yamamoto. Fumiki Katsuoka and Masayuki Yamamoto drafted the initial manuscript. All authors reviewed and approved the final manuscript.

### Conflicts of Interest

None

### Approval by Institutional Review Board

The Tohoku Medical Megabank Project was reviewed and approved by the ethics committees of both Tohoku University and Iwate Medical University. The Institutional Review Board approval numbers are 2012-4-564 for biobanking, 2012-4-565 and 2013-4-103 for cohorts, and 2020-4-191 for genome analysis.

### Data Availability

The data will be available via jMorp (https://jmorp.megabank.tohoku.ac.jp/) or will be made available upon request.
